# Perspectives of cancer patients and their health during the COVID-19 pandemic

**DOI:** 10.1371/journal.pone.0241741

**Published:** 2020-10-30

**Authors:** Emil Lou, Deanna Teoh, Katherine Brown, Anne Blaes, Shernan G. Holtan, Patricia Jewett, Helen Parsons, E. Waruiru Mburu, Lauren Thomaier, Jane Yuet Ching Hui, Heather H. Nelson, Rachel I. Vogel

**Affiliations:** 1 Division of Hematology, Oncology, and Transplantation, University of Minnesota, Minneapolis, Minnesota, United States of America; 2 Masonic Cancer Center, University of Minnesota, Minneapolis, Minnesota, United States of America; 3 Department of Obstetrics, Gynecology and Women’s Health, University of Minnesota, Minneapolis, Minnesota, United States of America; 4 Division of Health Policy and Management, School of Public Health, University of Minnesota, Minneapolis, Minnesota, United States of America; 5 Division of Epidemiology and Community Health, School of Public Health, University of Minnesota, Minneapolis, Minnesota, United States of America; 6 Department of Surgery, University of Minnesota, Minneapolis, Minnesota, United States of America; University of South Carolina, UNITED STATES

## Abstract

**Introduction:**

The immunosuppressive nature of some cancers and many cancer-directed treatments may increase the risk of infection with and severe sequelae from Coronavirus Disease 2019 (COVID-19). The objective of this study was to compare concerns about COVID-19 among individuals undergoing cancer treatment to those with a history of cancer not currently receiving therapy and to those without a cancer history.

**Methods:**

We conducted a cross-sectional anonymous online survey study of adults currently residing in the United States. Participants were recruited over a one-week period (April 3–11, 2020) using promoted advertisements on Facebook and Twitter. Groups were compared using chi-squared tests, Fisher’s exact tests, and t-tests.

**Results:**

543 respondents from 47 states provided information on their cancer history and were included in analyses. Participants receiving active treatment reported greater concern about infection from the SARS-CoV-2 coronavirus (p<0.001), higher levels of family distress caused by the COVID-19 pandemic (p = 0.004), and greater concern that the general public does not adequately understand the seriousness of COVID-19 (p = 0.04). Those with metastatic disease were more likely to indicate that COVID-19 had negatively affected their cancer care compared to patients with non-metastatic cancer (50.8% vs. 31.0%; p = 0.02). The most commonly reported treatment modifications included chemotherapy delays.

**Conclusions:**

Patients undergoing active treatment for cancer were most concerned about the short-term effects of the COVID-19 pandemic on the logistics as well as potential efficacy of ongoing cancer treatment, longer term effects, and overarching societal concerns that the population at large is not as concerned about the public health implications of SARS-CoV-2 infection.

## Introduction

The coronavirus disease 2019 (COVID-19) pandemic has led to sudden shifts in healthcare, including re-categorization of “essential” care [1]. The population of patients with a current or previous history of cancer represents a unique subset that as a whole may be more susceptible to SARS-CoV-2 coronavirus infection and sequelae of COVID-19 [2]. This is thought to be due to the immunosuppressive nature of some cancers and many cancer-directed treatments, and possibly due to more frequent clinical visits for patients undergoing active treatment and for those in active surveillance following treatment compared to patients without cancer [3, 4].

Under usual circumstances, a cancer diagnosis elicits anxiety regarding scheduling logistics, outcomes, and side-effects of cancer-directed treatments [5]. The recent changes in cancer care due to the COVID-19 pandemic [6], including treatment delays, cancellation of procedures deemed not essential, and a transition into virtual rather than in-person clinic visits, have likely added to these usual uncertainties and fears associated with having cancer.

We conducted a cross-sectional survey study to examine emotional well-being and health care decision-making in individuals with and without a history of cancer. We hypothesized that while distress and anxiety are increased in the general population related to the pandemic, these emotions would be higher in patients with a diagnosis of cancer. The primary objective of this survey study was to compare emotional well-being and decision-making among cancer patients undergoing therapy during the COVID-19 health crisis to two control groups: 1) cancer survivors who are not currently undergoing treatment and 2) those without a history of cancer.

## Methods

This anonymous cross-sectional online survey study was approved by the University of Minnesota Institutional Review Board. To be eligible, participants had to be 18 years of age or older, currently reside in the United States, and be able to read and write in English. Individuals were recruited over a one-week period (April 3–11, 2020) using promoted ads on the social media apps Facebook and Twitter, with separate ads specifically targeting those with a history of cancer. Invitations to participate in the survey were also posted by the American Cancer Society. Survey data were collected and stored using REDCap, a web-based data collection tool [7].

Survey items included demographics, personal concerns about COVID-19, and among those currently receiving cancer therapy, perceived effects of the COVID-19 pandemic on cancer treatment. Validated measures were used or modified as appropriate when possible. Symptoms of generalized anxiety and depression were measured using the General Anxiety Disorder-7 (GAD-7) [8] and Patient Health Questionnaire (PHQ-8) [9], respectively; and potentially clinically relevant cutoffs of 10 or greater were used for each. The number of COVID-19 cases in each state was determined using data from the Centers of Disease Control and Prevention (CDC) as of April 3, 2020 [10], and categorized as ≤1000 versus >1000 for analyses.

We used chi-squared tests and analysis of variance (ANOVA) to compare demographic characteristics between the three groups of interest: 1) cancer patients currently undergoing therapy, 2) individuals with a current or past diagnosis of cancer not currently undergoing therapy, 3) individuals with no history of cancer. The following demographic variables were categorized for analyses: gender (male, female, non-binary), race (white, black or African American, Asian, other), annual household income (<$50,000, $50,000-$99,999, ≥$100,000), education (no college degree, at least college degree), partner status (single, married/partnered), employment status (not working, working part or full time, retired). Descriptive statistics summarized rates of treatment plan changes among cancer patients currently receiving therapy, and compared those with and without metastatic disease using chi-squared and Fisher’s exact tests. Data were analyzed using SAS 9.4 (Cary, NC), and p-values <0.05 were considered statistically significant.

## Results

Among the 839 individuals who opened the survey, 815 (97%) were eligible for the study. A total of 763 answered at least one question and 543 participants provided sufficient information on their cancer history to be included in this analysis. Among eligible participants, 25.6% were undergoing active cancer treatment, 29.8% reported a history of cancer but no current active treatment, and 44.6% reported no history of cancer. Forty-seven states were represented; there were no respondents from Alaska, Vermont or Wyoming. Minnesota had the highest percentage of respondents (24.0%). Only two respondents reported being diagnosed with SARS-CoV-2. The average age of participants was 55.5 years (SD = 14.4), and the majority was female (82.8%) and non-Hispanic white (95.0%), with 23.9% being responsible for children under 18 years old, and 15.2% caring for other adult(s). Those with a history of cancer were older (p = 0.001), more likely to be retired (p = 0.003), and less likely to have a child under 18 years old living with them (p = 0.006; Table 1). Participants with cancer currently undergoing treatment were more likely to report their general health as fair or poor (p<0.001). All other demographic characteristics were balanced between the groups, including the number of COVID-19 cases in state of residence. Among those ever diagnosed with cancer, the three most common cancers were breast (40.5%), colon (16.6%), and non-melanoma skin cancer (10%). Approximately one-quarter (25.3%) of those with a cancer diagnosis reported stage IV cancer.

**Table 1:** Demographic characteristics.

	On active cancer treatment N = 139		History of cancer (not on active treatment) N = 162		No history of cancer N = 242		
**Characteristic**	**N**	**Mean±SD**	**N**	**Mean±SD**	**N**	**Mean±SD**	**p-value**
**Age, years**	127	56.3±12.3	145	59.1±12.3	226	52.8±16.1	<0.001
	**N**	**%**	**N**	**%**	**N**	**%**	**p-value**
**Gender**							0.40
Male	18	13.3	26	17.1	44	18.6	
Female	116	85.9	125	82.2	192	81.4	
Non-binary	1	0.7	1	0.7	0	0.0	
**Race**							0.69
White	126	94.0	150	97.4	218	94.0	
Black or African American	4	3.0	2	1.3	6	2.6	
Asian	2	1.5	2	1.3	4	1.7	
Other	2	1.5	0	0.0	4	1.7	
**Hispanic, Latino/a, or Spanish origin**							0.09
No	124	95.4	146	99.3	220	96.5	
Yes	6	4.6	1	0.7	8	3.5	
**Income**							0.52
<$50,000	38	28.8	38	25.0	62	26.6	
$50,000-$99,000	39	29.6	43	28.3	77	33.1	
≥100,000	34	25.8	48	31.6	71	30.5	
Prefer not to say	21	15.9	23	15.1	23	9.9	
**Education**							0.33
No college degree	55	40.7	68	44.2	114	48.5	
At least college degree	80	59.3	86	55.8	121	51.5	
**Partner Status**							0.96
Single	38	29.0	42	28.8	68	30.1	
Married / partnered	93	71.0	104	71.2	158	69.9	
**Employment status**							0.003
Not working	35	25.7	27	17.5	46	19.5	
Working part or full time	61	44.9	63	40.9	132	55.9	
Retired	40	29.4	64	41.6	58	24.6	
**Children under 18 living with you**							0.006
No	106	77.9	130	83.9	166	70.0	
Yes	30	22.1	25	16.1	71	30.0	
**Caring for adult**							0.01
No	124	91.2	133	86.4	190	80.2	
Yes	12	8.8	21	13.6	47	19.8	
**Urbanicity**							0.54
Rural	24	17.5	25	16.2	36	15.3	
Small city or town	54	39.4	48	31.2	82	34.9	
Suburb near large city	45	32.9	59	38.3	78	33.2	
Large city	14	10.2	22	14.3	39	16.6	
**COVID-19 Cases in Residing State (as of April 3, 2020)**							0.49
101–1000	47	34.6	59	39.9	90	40.7	
1001 or more	89	65.4	89	60.1	131	59.3	
**In general, would you say your health is**:							<0.001
Excellent or Very good	26	18.7	76	46.9	159	65.7	
Good	56	40.3	67	41.4	59	24.4	
Fair or Poor	57	41.0	19	11.7	24	9.9	

Rates of generalized anxiety and depression were similar across groups (Table 2). However, those in the active treatment group were more likely to report high levels of concern about getting infected by SARS-CoV-2 (71.9%), which was significantly higher than those who had completed cancer treatment (47.9%), or had no history of cancer (51.9%; p<0.001). In contrast, there was a gradient across groups when asked about their risk for a severe manifestation of the infection (p<0.001); only 33.8% of those with no cancer history felt they were at high risk for severe disease compared with 58.0% of those who had completed cancer treatment, and 90.7% of those who were in active treatment. Most respondents in the three groups reported that they regarded COVID-19 as a “moderately” or “very” serious threat, but patients with a history of previously treated cancer or cancer undergoing active treatment were more likely to report practicing complete social/physical distancing in the previous week as compared to individuals without cancer (p<0.001). Despite this difference, a majority of all three groups (>80%) reported high concern about close family members or friends becoming infected. Patients undergoing active treatment reported the highest level of family distress caused by the COVID-19 pandemic (p = 0.004), as well as concern that some others did not adequately understand the seriousness of COVID-19 and its implications (p = 0.04).

**Table 2:** Emotional health and COVID-19 experience and concerns.

	On active cancer treatment N = 139		History of cancer (not on active treatment) N = 162		No history of cancer N = 242		
**Variable**	**N**	**%**	**N**	**%**	**N**	**%**	**p-value**
**Generalized Anxiety**							0.49
No	99	73.9	122	78.7	174	73.7	
Yes	35	26.1	33	21.3	62	26.3	
**Depression**							0.07
No	96	73.3	125	82.2	171	72.5	
Yes	35	26.7	27	17.8	65	27.5	
**How concerned are about getting COVID-19?**							<0.001
Not at all or slightly concerned	15	11.1	31	19.6	64	26.7	
Somewhat concerned	23	17.0	45	28.5	61	25.4	
Moderately or extremely concerned	97	71.9	82	51.9	115	47.9	
**Consider yourself to be at "high risk" for severe illness from COVID-19?**							<0.001
No	7	5.0	32	19.8	120	50.0	
Yes	126	90.7	94	58.0	81	33.8	
Unsure	6	4.3	36	22.2	39	16.3	
**How serious do you think COVID-19 is?**							0.07
Not at all or a little serious	2	1.4	1	0.6	8	3.3	
Somewhat serious	2	1.4	5	3.1	14	5.8	
Moderately or very serious	135	97.1	156	96.3	220	90.9	
**How much have you been social distancing in the past week?**							<0.001
Not at all or a little	2	1.4	0	0.0	7	2.9	
Some or mostly	42	30.2	56	34.6	111	45.9	
Completely	95	68.4	106	65.4	124	51.2	
**How concerned about one of your close family members or friends getting COVID-19?**							0.07
Not at all or slightly concerned	12	8.6	4	2.5	22	9.1	
Somewhat concerned	15	10.8	15	9.3	23	9.5	
Moderately or extremely concerned	112	80.6	143	88.3	197	81.4	
**How concerned about getting needed healthcare if you become seriously ill from COVID-19?**							0.99
Not at all or slightly concerned	25	18.0	27	16.7	45	18.6	
Somewhat concerned	26	18.7	29	17.9	44	18.2	
Moderately or extremely concerned	88	63.3	106	65.4	153	63.2	
**How concerned about getting needed healthcare if you become ill from something else?**							0.75
Not at all or slightly concerned	32	23.0	36	22.2	65	26.9	
Somewhat concerned	31	22.3	31	19.1	47	19.4	
Moderately or extremely concerned	76	54.7	95	58.6	130	53.7	
	**N**	**Mean±SD**	**N**	**Mean±SD**	**N**	**Mean±SD**	**p-value**
**How distressing has COVID-19 been for your family?**	136	74.5±19.3	157	69.8±21.5	240	66.7±23.8	0.004
**Interference of COVID-19 with (self-) employment**	133	36.5±37.0	151	34.1±36.5	236	40.1±37.1	0.27
**Interference of COVID-19 with activities at home**	137	52.5±31.0	154	52.6±30.1	231	52.8±30.4	0.99
**Isolation related to COVID-19?**	138	74.0±26.5	156	68.0±28.7	236	67.7±26.5	0.08
**Financial burden from COVID-19 to date**	135	41.4±33.3	152	38.4±34.2	237	40.4±33.2	0.73
**Financial burden from COVID-19 over next 12 months**	138	53.6±31.4	154	51.0±33.0	237	54.2±33.0	0.62
**How worried about paying for medical care during the COVID-19 pandemic?**	137	36.6±34.7	151	31.5±33.9	233	36.4±33.7	0.33
**How concerned about some people not understanding the seriousness of COVID-19?**	137	89.0±16.1	158	83.9±21.3	234	83.9±21.7	0.04

Among those undergoing therapy, 20.7% overall reported no contact with their oncologists about treatment plans since the pandemic began. Those with metastatic cancer were significantly more likely to have had contact with their oncologist than those with non-metastatic disease (86.8% vs. 70.9%; p = 0.03) (Table 3). A higher proportion of participants with metastatic cancer reported that they “strongly” or “somewhat strongly” agreed with the statement that COVID-19 had negatively affected their cancer care, compared to participants with non-metastatic cancer (50.8% vs. 31.0%; p = 0.02).

**Table 3:** Comparison of cancer treatment concerns among those currently undergoing therapy or diagnosed with cancer during COVID-19 by cancer stage (metastatic vs. non-metastatic; N = 133).

	Non-metastatic		Metastatic		
Variable	N	%	N	%	p-value
**In contact with oncologist about treatment plan since COVID-19 pandemic began**					0.03
No	21	29.2	8	13.1	
Yes	51	70.8	53	86.9	
**The COVID-19 pandemic has negatively affected my cancer care**.					0.02
Strongly or somewhat agree	22	31.0	31	50.8	
Neutral/Somewhat disagree/Strongly disagree	49	69.0	30	49.2	
**Were your appointments moved to telephone visits or video encounters (telehealth)?**					0.59
Yes telephone always	33	64.7	28	52.8	
Yes telephone sometimes	2	3.9	1	1.9	
Yes video visits always	8	15.7	10	18.9	
Yes video visits sometimes	2	3.9	5	9.4	
Yes combination of telephone and video visits	1	2.0	4	7.6	
No	5	9.8	5	9.4	
**Has the COVID-19 health situation changed your treatment plan?**					0.80
No	51	70.8	41	67.2	
Yes	11	15.3	12	19.7	
Unsure	10	13.9	8	13.1	
**What role did you play in making that decision?**					0.41
I made the decision with little or no input from my doctor	5	8.6	4	7.0	
I made the decision after seriously considering my doctor’s opinion	3	5.2	6	10.5	
My doctor and I share responsibility for the decision together	32	55.2	31	54.4	
My doctor made the decision after seriously considering my opinion	4	6.9	8	14.0	
My doctor made the decision about my treatment with little or no input from me	14	24.1	8	14.0	
**How did your treatment plan change? (select all that apply)**					
Delayed surgery	3	4.2	2	3.3	1.00
Delayed chemotherapy infusion or other treatment	3	4.2	8	13.1	0.11
Delayed or altered radiation	2	2.8	0	0.0	0.50
Stopped chemotherapy earlier than planned	0	0.0	2	3.3	0.21
Other*	4	5.6	2	3.3	0.69
**What do you believe contributed to the decision to change your treatment plan? (select all that apply)**					
Concern about my COVID-10 exposure risk	7	63.6	12	100.0	0.04
Concern about availability of hospital beds and supplies	2	18.2	5	41.7	0.37
Concern about the availability of donated blood	2	18.2	1	8.3	0.59
Hospital/clinic rules related to COVID-19	5	45.5	4	33.3	0.68
Professional medical organization recommendations	0	0.0	1	8.3	1.00
Strict visitor policy	3	27.3	5	41.7	0.67
Transportation concerns	2	18.2	2	16.7	1.00

* delayed bone marrow transplant because international donor (n = 1), delayed scans (n = 2), delayed appointments (n = 1), changed care to local clinic instead of cancer center (n = 1)

Some (N = 26, 17.9%) of those currently receiving cancer therapy stated the pandemic changed their ongoing treatment plans. The most commonly specified form of treatment modification was delay or stopping of chemotherapy infusion (N = 14, 53.8%), followed by delayed surgery (N = 5, 19.2%). Concern about risk of exposure to COVID-19 was the most commonly cited reason for any changes (80.8%); other reasons included hospital or clinic policies (38.5%) or strict visitor policies (30.8%). Those who reported having treatment plans changed were more likely to report clinically relevant symptoms of anxiety (52.0% vs. 17.0%, p<0.001) and depression (56.0% vs. 20.0%, p<0.001) than those who did not report a treatment plan change.

Almost all participants noted a switch to at least some telehealth from in-person clinical visits. More than half of participants stated that decision-making was shared between the patient and physician; 7.2% reported that the decision was made “with little or no input from my doctor”, and conversely 19.2% reported that the decision was made “with little or no input from me.”

## Discussion

Patients with cancer undergoing active treatment had the highest level of concern of getting infected by SARS-CoV-2. More than 90% in active treatment feared having a severe manifestation of the infection. This same population reported the highest level of family distress caused by the COVID-19 pandemic, indicating that related anxiety extended beyond just individual concern. However, the increased extent of anxiety was focused on COVID-19 and potential complications related to this infection. As the self-reported prevalence of generalized anxiety and depression did not differ significantly between patients actively being treated for cancer vs. respondents with no history of cancer, it is possible that the societal level of anxiety overall was high during the onset of the pandemic, independent of cancer diagnosis, and this may have accounted for that lack of difference.

Nearly 20% of participants reported that treatment changes during the pandemic were done “with little or no input” from them, but rather the logistical changes of cancer care were initiated exclusively or nearly exclusively by their treatment team. The full impact of the COVID-19 pandemic on cancer patients may not be known for years, if not decades. In the immediate short-term, the current situation has rapidly altered the landscape of acceptable modifications to treatment in order to most effectively balance safety with efficacy of treatment. What we learned from this study is that, whether in fact or by patient perception, the extent of engagement of cancer-treating teams did not match the height of anxiety that many patients with cancer had regarding their own health and also the health of their family members during the onset and peak of this pandemic. We can use this information to better educate the medical community that patients need firm guidance and communication early and often during such times of crisis; this point is especially important as we now anticipate additional ‘waves’ of peaks of the COVID-19 pandemic, and cannot rule out future pandemics from other causes. Initial efforts to quickly adapt cancer care to the new environment prioritizing safety focused mostly on discussions among medical experts; these results shine a spotlight on the equally important need to prioritize community engagement with the patient advocacy community and support groups to disseminate needed reassurance, in addition to conveying these changes to patients directly.

Individual partnerships between patient and physician are integral and crucial to achieving this goal. The major concerns noted by respondents in our survey align with those reported on April 17, 2020 in the American Cancer Society’s (ACS) Findings Summary from its recent advocacy survey on the pandemic’s impact on cancer patients and survivors [11]. Key findings from that survey included 50% of cancer patients and survivors reporting impact on their healthcare during this time, with 27% of all patients who had been undergoing active treatment reporting some delay. Similar to our survey, 40% of ACS respondents undergoing active treatment expressed acute concerns about effects on their cancer-directed therapy plans [11].

Our survey identifies some important gaps in knowledge. Specifically, what risks are posed to those that have a history of cancer but have completed treatment and are in less frequent contact with their care team? This group perceived their risk for contracting SARS-CoV-2 as being notably lower than those on active treatment. However, they also were concerned that they were at higher risk for developing severe disease. How susceptibility and disease pathogenesis are impacted by prior cancer therapy is a pressing research question, given the large number of survivors who have completed treatment, and accelerated aging and increased prevalence of cardiovascular disease that often accompany chemotherapeutic treatments.

One strength of this study was harnessing social media to quickly disseminate a research study in a short period of time. However, limitations of this method led to disproportionate representation of cancer patients residing in Minnesota and the Midwest, thus limiting generalizability of study results to all cancer patients and the general population. The majority of respondents was skewed toward a specific (>90% white and >80% female) demographic that also represented a technologically savvy population familiar and at ease with use of social media and associated technology. A more diverse population with regards to race and sex would have been more representative of the population of patients with cancer in the U.S. and may have provided different results. Furthermore, the heterogeneity of cancer types represented, with inherent differences in treatment strategies and effect of treatment modifications and delays, impacts the ability to report specific alterations in treatment plans and the effect of specific management alterations on patient emotional state. Another limitation is the inability to discern between perception or misperception of patients on whether their cancer care was negatively affected by early changes to routine administration of treatment, in efforts to maximize patient safety. Some general examples of such changes included modifications to dosages and frequency of administration of systemic chemotherapies at a time when the duration and severity of COVID-19 were not immediately clear. Recent and future studies objectively examining changes in patterns of cancer diagnoses and outcomes in the years to come will help to clarify this issue.

In summary, patient respondents undergoing active treatment for cancer harbored concerns about short-term effects of the COVID-19 pandemic on the logistics and potential efficacy of ongoing cancer treatment, about longer term effects, and that the population at large did not take the public health implications of the SARS-CoV-2 virus seriously enough. The cancer care landscape is quickly evolving to meet the needs of patients by attempting to balance safety with treatment efficacy. Over the long term, some of these adaptations, including virtual visits, may be permanently adopted by patients and healthcare systems as integral to overall cancer care. Regardless of which approaches will be taken, partnership and adequate levels of communication between cancer care teams and patients with cancer are and will continue to remain critical during and for a long time following the current pandemic.

## Supporting information

S1 File COVID-19 perceptions of health survey. (PDF)
