# Supplementary material for: Perspectives of cancer patients and their health during the COVID-19 pandemic
Source: PLoS One. 2020 Oct 30;15(10):e0241741. doi: 10.1371/journal.pone.0241741 (PMC7598454; doi:10.1371/journal.pone.0241741)
Supplement: S1 File — (PDF) [file pone.0241741.s001.pdf]

COVID-19 Perceptions of Health Survey

Perceptions of COVID-19 and Health

You are invited to be in a research study to understand how the COVID-19 health crisis is affecting the health and well-being of people in the United States. You were identified as a possible participant because you responded to a post or ad on Twitter or Facebook. We ask that you read the following description of the study and ask any questions you may have before agreeing to participate in the study.

This study is being conducted by Rachel I. Vogel, PhD, University of Minnesota, Department of Obstetrics, Gynecology and Women's Health.

Procedures:
If you agree to be in this study, we would ask you to take an online, anonymous survey which will take you about 10-15 minutes to complete.

Confidentiality:
We will not be collecting any identifying or contact information. The records of this study will be kept private. In any sort of report we might publish, we will not include any information that will make it possible to identify a subject. Research records will be stored securely and only researchers will have access to the records.

Voluntary Nature of the Study:
Participation in this study is voluntary. You may skip any questions you do not feel comfortable answering. Your decision whether or not to participate will not affect your current or future relations with the University of Minnesota. If you decide to participate, you are free to not answer any question or withdraw at any time without affecting those relationships.

Contacts and Questions:
The researcher conducting this study is: Rachel I. Vogel, Ph.D. If you have questions later, you are encouraged to contact them at the University of Minnesota, 420 Delaware Street SE MMC 395, Minneapolis, MN 55455, phone: 612-624-6928, email: UMNCancerSurv@umn.edu.

This research has been reviewed and approved by an IRB within the Human Research Protections Program (HRPP). To share feedback privately with the HRPP about your research experience, call the Research Participants' Advocate Line at 612-625-1650 (Toll Free: 1-888-224-8636) or go to z.umn.edu/participants. You are encouraged to contact the HRPP if:

- Your questions, concerns, or complaints are not being answered by the research team.
- You cannot reach the research team.
- You want to talk to someone besides the research team.
- You have questions about your rights as a research participant.
- You want to get information or provide input about this research.

You can download a copy of this information to keep for your records.

[Attachment: "Information_Sheet_03302020_clean.docx"]

Please confirm you are eligible for this study before proceeding:

Are you 18 years old or older?

◯ Yes
◯ No

Do you currently live in the United States?

◯ Yes
◯ No

projectredcap.org

Can you read and write in English?

◯ Yes
◯ No

These first questions will ask you general questions about your health.

In general, would you say your health is:

◯ Excellent
◯ Very good
◯ Good
◯ Fair
◯ Poor

Please select the number (0-10) that best describes how much distress you have been experiencing in the past week including today.

◯ 0=No Distress
◯ 1
◯ 2
◯ 3
◯ 4
◯ 5
◯ 6
◯ 7
◯ 8
◯ 9
◯ 10=Extreme Distress

Please indicate the extent to which you agree with each of the statements below:

	Strongly Disagree	Disagree	Neutral	Agree	Strongly Agree
I tend to bounce back quickly after hard times.	○	○	○	○	○
I have a hard time making it through stressful events.	○	○	○	○	○
It does not take me long to recover from a stressful event.	○	○	○	○	○
It is hard for me to snap back when something bad happens.	○	○	○	○	○
I usually come through difficult times with little trouble	○	○	○	○	○
I tend to take a long time to get over set-backs in my life.	○	○	○	○	○

Over the last 2 weeks, how often have you been bothered by any of the following problems?

	Not at all	Several days	More than half of the days	Nearly every day
Little interest or pleasure in doing things	◯	◯	◯	◯
Feeling down, depressed, or hopeless	◯	◯	◯	◯
Trouble falling or staying asleep, or sleeping too much	◯	◯	◯	◯
Feeling tired or having little energy	◯	◯	◯	◯
Poor appetite or overeating	◯	◯	◯	◯
Feeling bad about yourself--or that you are a failure or have let yourself or your family down	◯	◯	◯	◯
Trouble concentrating on things, such as reading the newspaper or watching television	◯	◯	◯	◯
Moving or speaking so slowly that other people could have noticed. Or the opposite-being so fidgety or restless that you have been moving around a lot more than usual	◯	◯	◯	◯

How difficult have these problems made it for you to do your work, take care of things at home, or get along with other people?

◯ Not difficult at all
◯ Somewhat difficult
◯ Very difficult
◯ Extremely difficult

Over the last 2 weeks, how often have you been bothered by the following?

	Not at all	Several days	Over half the days	Nearly every day
Feeling nervous, anxious or on edge	◯	◯	◯	◯
Not being able to sleep or control worrying	◯	◯	◯	◯
Worrying too much about different things	◯	◯	◯	◯
Trouble relaxing	◯	◯	◯	◯
Being so restless that it's hard to sit still	◯	◯	◯	◯
Becoming easily annoyed or irritable	◯	◯	◯	◯
Feeling afraid as if something awful might happen	◯	◯	◯	◯

How difficult have these problems made it for you to do your work, take care of things at home, or get along with other people?

◯ Not difficult at all
◯ Somewhat difficult
◯ Very difficult
◯ Extremely difficult

The following questions are specifically related to COVID-19 (Coronavirus 2019).

Because of the COVID-19 pandemic, recommendations have been made that individuals reduce their contact with others, by working remotely, self-isolating at home, keeping their children out of school, keeping a physical distance from each other, and through other measures to minimize in-person contacts between people. These contact-reducing measures have been called 'social distancing'.

How much have you been social distancing in the past week?

◯ Not at all
◯ A little
◯ Some
◯ Mostly
◯ Completely

Which of the following have you done in the past week? Check all that apply.

☐ Cancelled travel plans
☐ Stopped going out to eat at restaurants
☐ Worked from home
☐ Avoided large groups or crowded spaces
☐ Visited loved ones by phone or video rather than in person
☐ Didn't leave my house/apartment/flat/property
☐ Changed the way I go shopping (e.g. other people now go shopping for me, switched to online shopping)
☐ Cancelled non-emergent medical appointments
☐ Other

Describe what other social distancing you have done.

____

Have you experienced any of the following symptoms in the past month? Check all that apply.

☐ Cough
☐ Fever
☐ Sore Throat
☐ New/sudden fatigue or tiredness
☐ Difficulty breathing
☐ Change in sense of taste or smell
☐ None

Have you been directly exposed to someone with COVID-19 in the past month?

◯ Yes
◯ No
◯ Unsure

Have you been diagnosed with COVID-19 in the past month?

◯ Yes - confirmed with a positive test
◯ Yes - presumed
◯ No - tested negative
◯ No - not tested
◯ Unsure

Do you know how/where you were exposed?

◯ Personal international travel
◯ Close contact with someone who travelled internationally
◯ Personal domestic travel (within the United States)
◯ Close contact with someone who travelled domestically (within the United States)
◯ Hospital or clinic
◯ Community contact
◯ Unknown

What interactions with the healthcare system did you have related to COVID-19? Choose all that apply.

☐ None, isolated at home
☐ Virtual or telephone health provider visit
☐ Drive-up testing
☐ In-person health provider visit (general physician or urgent care)
☐ Emergency room
☐ Hospitalization
☐ Ventilation/Intensive Care Unit (ICU)

How concerned are you about getting COVID-19?

◯ Not at all concerned
◯ Slightly concerned
◯ Somewhat concerned
◯ Moderately concerned
◯ Extremely concerned

Do you consider yourself to be at "high risk" for severe illness from COVID-19?

◯ No
◯ Yes
◯ Unsure

Indicate why you consider yourself to be at "high risk" for severe illness from COVID-19. Check all that apply.

☐ Older adult (>60 years old)
☐ History of cancer (completed active cancer treatment prior to January 2020)
☐ Immune-compromised (ex. HIV, bone marrow transplant, organ transplant, chemotherapy, taking immune-supressing drugs for Crohn's disease or rheumatoid arthritis)
☐ Heart disease
☐ Diabetes
☐ Lung disease (e.g. asthma, emphysema/COPD)
☐ Pregnant
☐ Active cancer treatment other than chemotherapy (recent surgery, recent/ongoing radiation therapy, recent/ongoing endocrine therapy)
☐ Obesity
☐ Active smoker
☐ Previous/Former smoker
☐ Other reason

Please specify

____

Is there someone (beside yourself) living with you that you consider to be at "high risk" for severe illness from COVID-19?

◯ No
◯ Yes
◯ N/A (I live alone)
◯ Unsure

How serious do you think COVID-19 is?

◯ Not at all serious
◯ A little serious
◯ Somewhat serious
◯ Moderately serious
◯ Very serious

How concerned are you about one of your close family members or friends getting COVID-19?

◯ Not at all concerned
◯ Slightly concerned
◯ Somewhat concerned
◯ Moderately concerned
◯ Extremely concerned
◯ Not applicable

How concerned are you about getting the healthcare you need if you become seriously ill from COVID-19?

◯ Not at all concerned
◯ Slightly concerned
◯ Somewhat concerned
◯ Moderately concerned
◯ Extremely concerned

How concerned are you about getting the healthcare you need if you become ill from something other than COVID-19?

◯ Not at all concerned
◯ Slightly concerned
◯ Somewhat concerned
◯ Moderately concerned
◯ Extremely concerned

Did you get the influenza (flu) vaccine this season (2019-2020)?

◯ No
◯ Yes
◯ Unsure

How likely are you to choose to get a vaccine for COVID-19 if it is available in the next year?

◯ Not at all likely
◯ Slightly likely
◯ Somewhat likely
◯ Moderately likely
◯ Very likely

How distressing has COVID-19 been for your family?

0=Not at all   100=A great deal

(Place a mark on the scale above)

To what degree has COVID-19 interfered with your employment, including self-employment?

0=No problem — 100=Severe problem

(Place a mark on the scale above)

To what degree has COVID-19 interfered with your activities at home?

0=No problem — 100=Severe problem

(Place a mark on the scale above)

How much isolation do you feel related to COVID-19?

0=Not at all — 100=A great deal

(Place a mark on the scale above)

How much financial burden have you incurred as a result of COVID-19?

0=Not at all — 100=A great deal

(Place a mark on the scale above)

How much financial burden do you think you will have incurred as a result of COVID-19 over the next 12 months?

0=Not at all — 100=A great deal

(Place a mark on the scale above)

How worried are you that you will not be able to pay for medical care during the COVID-19 pandemic?

0=Not at all — 100=A great deal

(Place a mark on the scale above)

How concerned are you that some people do not understand the seriousness of COVID-19?

0=Not at all — 100=A great deal

(Place a mark on the scale above)

How has the COVID-19 pandemic affected your employment status (including self-employment or any business that you may be a part of)? Select all that apply.

- ☐ No change
- ☐ Changed to tele-work arrangement (work from home)
- ☐ Increased working hours
- ☐ Reduced working hours
- ☐ Increased pay
- ☐ Decreased pay
- ☐ Laid off
- ☐ Close business temporarily
- ☐ Other

Please specify.

____

As a result of COVID-19, have you eaten less than you felt you should because there wasn't enough money for food?

◯ Yes
◯ No

Are you covered by any form of health insurance or health plan?

◯ Yes, I am covered by health insurance
◯ No, I am not covered by health insurance
◯ I don't know

Which of the following is your MAIN source of health insurance coverage?

◯ Plan through your employer
◯ Plan through your spouse's employer
◯ Plan you purchased yourself
◯ Medicare
◯ Medicaid
◯ Plan through your parents/mother/father
◯ Somewhere else
◯ I don't know

As a result of COVID-19, has the electric, gas, oil, or water company threatened to shut off services in your home?

◯ Yes
◯ No
◯ Not applicable

As a result of COVID-19, are issues with child care making it difficult for you to work or study?

◯ Yes
◯ No
◯ Not applicable

In this survey, we have mostly focused on potential negative consequences of the COVID-19 pandemic on your life. However, there may also be some positive consequences of the pandemic for people's lives and lifestyles.

In the past 2 weeks, have you noticed any changes in your everyday life and lifestyle that you think are positive compared with the time before the COVID-19 outbreak?

These next questions ask about sources of information about COVID-19 (coronavirus).

In the past week, how much have you heard about COVID-19 from any media source (e.g., the internet, television, radio or podcasts, newspapers)?

◯ None
◯ A little
◯ Some
◯ A lot

Where are you getting most of your news about COVID-19?

◯ On television
◯ On news websites or apps
◯ Through social networking sites (such as Facebook or Twitter)
◯ On the radio
◯ In print

Thinking now about specific information sources, which of the following has been your primary source of information on COVID-19?

◯ Fox News
◯ CNN
◯ MSNBC
◯ Local TV
◯ National newspaper
◯ Local newspaper
◯ National radio
◯ Local radio
◯ Google or Yahoo
◯ Social media (ex. Facebook, Twitter)
◯ Other

Please specify. __________________________________

About how often have you talked with family or friends about COVID-19 (coronavirus) in the past week?

◯ Not at all
◯ About once per week
◯ A few times a week
◯ Daily

These next questions are about your health history.

Has a medical doctor or healthcare professional EVER told you that you have any of the following?

	Yes	No
Heart problems, such as a heart attack, coronary artery disease, congestive heart failure, irregular heartbeat, etc.	○	○
Hypertension (high blood pressure)	○	○
Chronic back pain, including sciatica (pain or numbness that travels down your leg to below your knees)	○	○
Arthritis	○	○
Stroke, including mini-stroke or blood clots in the brain	○	○
Severe problems with memory or concentration	○	○
Asthma, emphysema, or COPD (Chronic Obstructive Pulmonary Disease)	○	○
Stomach and/or intestinal problems, such as Crohn's disease, ulcers, or inflammatory bowel disease	○	○
Diabetes, high blood sugar, or sugar in urine	○	○
Depression (feeling sad or blue) that resulted in treatment with medication and/or counseling	○	○
Anxiety or "nervousness" that resulted in treatment with medication and/or counseling	○	○
Neuropathy (numbness in both hands or both feet)	○	○
Other chronic condition	○	○

Please specify.

____

Are you a current smoker?

◯ Yes
◯ No - never smoker
◯ No - previous smoker

Have you ever used an electronic cigarette/e-cig or vaped (with or without nicotine)?

◯ Yes
◯ No
◯ I don't know

Have you ever been diagnosed with cancer?

◯ Yes
◯ No
◯ Unsure

What type of cancer did you have? Check all that apply.

☐ Bladder cancer
☐ Bone cancer
☐ Breast cancer
☐ Cervical cancer
☐ Colon cancer
☐ Endometrial/Uterine cancer
☐ Head/Neck cancer
☐ Hodgkins
☐ Renal cancer
☐ Leukemia
☐ Liver cancer
☐ Lung cancer
☐ Melanoma
☐ Non-Hodgkins
☐ Oral cancer
☐ Ovarian cancer
☐ Pancreatic cancer
☐ Pharyngeal cancer
☐ Prostate cancer
☐ Rectal cancer
☐ Sarcoma
☐ Skin cancer (not melanoma)
☐ Other

Please specify

____

Were you diagnosed with cancer during COVID-19 pandemic (since February 2020)?

◯ Yes
◯ No
◯ Unsure

Did you have problems accessing health care providers to initiate cancer treatment?

◯ Yes
◯ No
◯ Unsure

What treatment have you received for your cancer? Check all that apply.

☐ None
☐ Surgery
☐ Chemotherapy (IV or pills)
☐ Radiation
☐ Immunotherapy
☐ Bone Marrow Transplant (BMT)
☐ Endocrine therapy (hormone therapy)
☐ Other

Please specify.

____

How long ago did you finish your most recent treatment?

◯ Still receiving treatment
◯ During the past year
◯ 1-2 years ago
◯ 2-3 years ago
◯ 3-4 years ago
◯ 4-5 years ago
◯ Over 5 years ago

Do you currently have metastatic (stage IV) disease?

◯ Yes
◯ No
◯ Unsure

Have you been in contact with your oncologist about your treatment plan since COVID-19 health situation began?

◯ Yes
◯ No
◯ Unsure

Were your appointment moved to telephone visits or video encounters (telehealth)?

◯ Yes - telephone always
◯ Yes - telephone sometimes
◯ Yes - video visits always
◯ Yes - video visits sometimes
◯ Yes - Combination of telephone and video visits
◯ No

Has the COVID-19 health situation changed your treatment plan?

◯ Yes
◯ No
◯ Unsure

How did your treatment plan change? Select all that apply.

- ☐ Delayed surgery
- ☐ Delayed chemotherapy infusion
- ☐ Delayed radiation therapy
- ☐ Stopped chemotherapy earlier than planned
- ☐ Stopped radiation earlier than planned'
- ☐ Altered sequence of treatment (e.g. started with chemotherapy instead of surgery)
- ☐ Moved surgery up/sooner than originally planned
- ☐ Other

Please specify

____

What role did you play in making that decision?

- ◯ I made the decision with little or no input from my doctor
- ◯ I made the decision after seriously considering my doctor's opinion
- ◯ My doctor and I share responsibility for the decision together
- ◯ My doctor made the decision after seriously considering my opinion
- ◯ My doctor made the decision about my treatment with little or no input from me

Which of the following do you believe contributed to the decision to change your treatment plan? Choose all that apply.

- ☐ Concern about my COVID-19 exposure risk
- ☐ Concern about availability of hospital supplies and beds
- ☐ Concern about the availability of donated blood
- ☐ Hospital/clinic rules related to COVID-19
- ☐ Professional medical organization recommendations
- ☐ Closure of patient and family accommodations (such as American Cancer Society Hope Lodge)
- ☐ Strict visitor policy
- ☐ Transportation concerns due to suspension of medical transportation services
- ☐ Other

Please describe.

To what extent do you agree with the following statement: The COVID-19 pandemic has negatively affected my cancer care.

- ◯ Strongly agree
- ◯ Somewhat agree
- ◯ Neutral
- ◯ Somewhat disagree
- ◯ Strongly disagree

How much financial burden have you incurred related to your cancer treatment as a result of COVID-19?

0=Not at all 100=A great deal

(Place a mark on the scale above)

Have you delayed any cancer screening appointments or procedures (e.g. mammogram, colonoscopy, Pap smear, etc)?

- ◯ Yes
- ◯ No
- ◯ Not applicable
- ◯ Unsure

Which screening or procedure did you delay? Select all that apply.

- ☐ Colonoscopy
- ☐ Pap smear/HPV test
- ☐ Mammogram
- ☐ Breast MRI
- ☐ Risk-reducing mastectomy (removal of breasts)
- ☐ Risk-reducing oophorectomy (removal of ovaries)
- ☐ Other

Please specify.

____

These last questions ask about your demographic characteristics.

What is your current age?

____

What is your biologic sex (sex/gender assigned at birth)?

◯ Male
◯ Female
◯ Intersex
◯ Prefer not to answer

Do you identify as transgender, gender-queer, gender-fluid, or another non-binary gender?

◯ Yes
◯ No
◯ I don't know
◯ Prefer not to answer

What is your race? Select all that apply?

☐ White
☐ Black or African American
☐ American Indian or Alaska Native
☐ Asian Indian
☐ Chinese
☐ Filipino
☐ Japanese
☐ Korean
☐ Vietnamese
☐ Other Asian
☐ Native Hawaiian
☐ Guamanian or Chamorro
☐ Samoan
☐ Other Pacific Islander

Are you Hispanic, Latino/a, or of Spanish origin? Select all that apply.

☐ No, not of Hispanic, Lation/a, Spanish origin
☐ Yes, Mexican, Mexican American, Chicano/a
☐ Yes, Puerto Rican
☐ Yes, Cuban origin

How tall are you?

Feet

◯ 4
◯ 5
◯ 6
◯ 7

Inches

◯ 0
◯ 1
◯ 2
◯ 3
◯ 4
◯ 5
◯ 6
◯ 7
◯ 8
◯ 9
◯ 10
◯ 11

What is your current weight (pounds/lbs)?

____

What is the highest level of schooling you have completed?

◯ Less than high school
◯ High school graduate
◯ Vocational or business school or AA degree
◯ Some college
◯ College or university graduate (bachelor's degree)
◯ Graduate or professional training (graduate degree)

Are you a healthcare worker?

◯ Yes
◯ No

Which category best describes your role?

◯ Physician
◯ Nurse (RN,NP)
◯ Physician Assistant
◯ Pharmacist
◯ Technologist or Technician
◯ Therapist
◯ Administrative staff
◯ Environmental services
◯ Other

Please specify.

____

What is your current partner status?

◯ Single / no partner
◯ Married / Partnered and live together
◯ Married / Partnered but do not live together
◯ Other

Do you have children under the age of 18 living with you and/or under your care?

◯ Yes
◯ No

Are you currently involved in caring for an older adult?

◯ Yes
◯ No

Are you currently employed or self-employed?

◯ Yes - Full Time
◯ Yes - Part Time
◯ No
◯ Retired

What is your annual household income?

◯ Less than $20,000
◯ $20,000 to $49,999
◯ $50,000 to $74,999
◯ $75,000 to $99,999
◯ $100,000 to $149,999
◯ $150,000 to $199,999
◯ $200,000 or more
◯ Prefer not to say

What state/territory are you currently residing in?

- ◯ Alabama (AL)
- ◯ Alaska (AK)
- ◯ Arizona (AZ)
- ◯ Arkansas (AR)
- ◯ California (CA)
- ◯ Colorado (CO)
- ◯ Connecticut (CT)
- ◯ Delaware (DE)
- ◯ District of Columbia (DC)
- ◯ Florida (FL)
- ◯ Georgia (GA)
- ◯ Hawaii (HI)
- ◯ Idaho (ID)
- ◯ Illinois (IL)
- ◯ Indiana (IN)
- ◯ Iowa (IA)
- ◯ Kansas (KS)
- ◯ Kentucky (KY)
- ◯ Louisiana (LA)
- ◯ Maine (ME)
- ◯ Maryland (MD)
- ◯ Massachusetts (MA)
- ◯ Michigan (MI)
- ◯ Minnesota (MN)
- ◯ Mississippi (MS)
- ◯ Missouri (MO)
- ◯ Montana (MT)
- ◯ Nebraska (NE)
- ◯ Nevada (NV)
- ◯ New Hampshire (NH)
- ◯ New Jersey (NJ)
- ◯ New Mexico (NM)
- ◯ New York (NY)
- ◯ North Carolina (NC)
- ◯ North Dakota (ND)
- ◯ Ohio (OH)
- ◯ Oklahoma (OK)
- ◯ Oregon (OR)
- ◯ Pennsylvania (PA)
- ◯ Rhode Island (RI)
- ◯ South Carolina (SC)
- ◯ South Dakota (SD)
- ◯ Tennessee (TN)
- ◯ Texas (TX)
- ◯ Utah (UT)
- ◯ Vermont (VT)
- ◯ Virginia (VA)
- ◯ Washington (WA)
- ◯ West Virginia (WV)
- ◯ Wisconsin (WI)
- ◯ Wyoming (WY)
- ◯ American Samoa (AS)
- ◯ Guam (GU)
- ◯ Northern Mariana Islands (MP)
- ◯ Puerto Rico (PR)
- ◯ Virgin Islands (VI)

What type of community do you live in?

◯ Rural area
◯ Small city or town
◯ Suburb near a large city
◯ Large city

In general, do you think of yourself as...

◯ Extremely liberal
◯ Liberal
◯ Slightly liberal
◯ Moderate, middle of the road
◯ Slightly conservative
◯ Conservative
◯ Extremely conservative

Generally speaking, do you think of yourself as a...

◯ Republican
◯ Democrat
◯ Independent
◯ Another party
◯ No preference

Please specify.

____
